# Bypassing the Need for the Transcriptional Activator EarA through a Spontaneous Deletion in the BRE Portion of the *fla* Operon Promoter in *Methanococcus maripaludis*

**DOI:** 10.3389/fmicb.2017.01329

**Published:** 2017-07-17

**Authors:** Yan Ding, Alison Berezuk, Cezar M. Khursigara, Ken F. Jarrell

**Affiliations:** ^1^Department of Biomedical and Molecular Sciences, Queen’s University, Kingston ON, Canada; ^2^Department of Molecular and Cellular Biology, University of Guelph, Guelph ON, Canada

**Keywords:** BRE deletion, archaellum, EarA, promoter, *fla* operon, archaea

## Abstract

In *Methanococcus maripaludis*, the euryarchaeal archaellum regulator A (EarA) is required for the transcription of the *fla* operon, which is comprised of a series of genes which encode most of the proteins needed for the formation of the archaeal swimming organelle, the archaellum. In mutants deleted for *earA* (Δ*earA*), there is almost undetectable transcription of the *fla* operon, Fla proteins are not synthesized and the cells are non-archaellated. In this study, we have isolated a spontaneous mutant of a Δ*earA* mutant in which the restoration of the transcription and translation of the *fla* operon (using *flaB2*, the second gene of the operon, as a reporter), archaella formation and swarming motility were all restored even in the absence of EarA. Analysis of the DNA sequence from the *fla* promoter of this spontaneous mutant revealed a deletion of three adenines within a string of seven adenines in the transcription factor B recognition element (BRE). When the three adenine deletion in the BRE was regenerated in a stock culture of the Δ*earA* mutant, very similar phenotypes to that of the spontaneous mutant were observed. Deletion of the three adenines in the *fla* promoter BRE resulted in the mutant BRE having high sequence identity to BREs from promoters that have strong basal transcription level in *Mc. maripaludis* and *Methanocaldococcus jannaschii*. These data suggest that EarA may help recruit transcription factor B to a weak BRE in the *fla* promoter of wild-type cells but is not required for transcription from the *fla* promoter with a strong BRE, as in the three adenine deletion version in the spontaneous mutant.

## Introduction

In the third domain of life, the Archaea, the transcription machinery is composed of a multi-subunit RNA polymerase that shares homology to the eukaryotic RNA polymerase II, as well as two general transcription factors: the TATA-box binding protein (TBP) and transcription factor B (TFB) (Bell and Jackson, 2001; Jun et al., 2011; Gehring et al., 2016). The corresponding DNA elements of a basal archaeal promoter includes a purine-rich transcription factor B recognition element (BRE), which is recognized by the TFB, immediately followed by a TATA box centered at a distance of 26/27 bp upstream of the transcription start site (TSS) (Soppa, 1999; Bartlett, 2005; Gehring et al., 2016). To initiate transcription, TBP first binds to TATA box. This is followed by the binding of TFB to the DNA-TBP complex by recognition of the BRE sequence (Bell et al., 1999) and, finally, the recruitment of RNA polymerase to initiate transcription (Bell and Jackson, 2001). Mutations in either the TATA box or BRE can decrease transcription levels by reducing recruitment of TBP and TFB (Bartlett, 2005).

Although Archaea use a eukaryote-like basal transcription machinery, the genome structure and its transcription regulation are more like that found in Bacteria. In Archaea, a cluster of genes is co-transcribed into a poly-cistronic mRNA under the control of a single promoter, which can be regulated by repressors and/or activators (Peeters et al., 2013). Transcriptional activators typically bind to sites located upstream of the BRE and help in the recruitment of TBP or TFB. In contrast, repressors can bind to either the promoter region where they interfere with TFB or TBP binding by steric hindrance, or downstream of the promoter, sometimes even after the TSS, to prevent RNA polymerase recruitment or transcription elongation (Bell, 2005; Peeters et al., 2013; Karr, 2014). Transcriptional activators are often associated with promoters that have TATA box or BRE sequences that deviate from consensus sequences (Ochs et al., 2012; Peeters et al., 2013). They are believed to help overcome poor binding of TBP or TFB to weak TATA and BRE sequences to activate transcription (Ouhammouch et al., 2003; Peng et al., 2009; Ochs et al., 2012).

The methanogen *Methanococcus maripaludis* is a member of the phylum Euryarchaeota and a model organism for studies in Archaea. Here, the *fla* operon, encoding the components of the archaeal swimming organelle, the archaellum (Jarrell and Albers, 2012; Albers and Jarrell, 2015), begins with *flaB1-B3* encoding the three major structural proteins (archaellins), followed by the *fla*-associated genes *flaC-J* (Chaban et al., 2007). Transcription of the *fla* operon is controlled by the transcriptional activator EarA (Ding et al., 2016b). Deletion of *earA* results in almost undetectable transcription of the *fla* operon and a corresponding disappearance of FlaB2 protein and archaella production (Ding et al., 2016b). Immediately upstream of the BRE in the *fla* promoter, four 6 bp consensus sequences were identified as EarA binding sites. When all four EarA binding sites were eliminated in the genome of wild-type *Mc. maripaludis*, similar phenotypes were observed as in the Δ*earA* mutant (Ding et al., 2016b). Recently, we have shown that EarA homologs from selected archaellated methanogens could successfully complement the function of EarA in the *Mc. maripaludis* Δ*earA* mutant, indicating that the EarA regulatory model is likely widespread in the methanogen *fla* promoters (Ding et al., 2017).

In addition to the direct control of transcription of the *fla* operon by EarA, transcription of the *fla* operon was also found to be regulated under several growth conditions. Global transcriptome analysis of *Mc. maripaludis* showed that the transcription of the *fla* operon is up-regulated when H_2_ is limited and down-regulated under leucine starvation, for example (Hendrickson et al., 2008). In addition, we recently showed that transcription of the *fla* operon was severely impaired in cells grown at temperatures greater than 38°C (Ding et al., 2016a). The mechanism behind the regulation of the *fla* promoter under the above conditions, including any possible involvement of EarA or other putative transcriptional activators or repressors, is yet to be reported.

In this study, we isolated a spontaneous mutant of the Δ*earA* mutant in which transcription of the *fla* operon, production of archaellins and archaellation were all restored to near wild-type levels, despite the absence of EarA. Analysis of the *fla* promoter region of this mutant revealed a deletion of three adenines in the BRE. Recreation of the three adenine deletion in the original Δ*earA* mutant by molecular biology techniques resulted in very similar archaella-related phenotypes as observed in the spontaneous mutant. Examination of the *fla* promoter wild-type BRE and the three adenine deletion BRE revealed that the mutant BRE were highly similar to BRE sequences associated with promoters with strong basal transcription levels in both *Mc. maripaludis* and a related hyperthermophilic methanogen *Methanocaldococcus jannaschii*.

## Materials and Methods

### Strains and Culture Conditions

*Methanococcus maripaludis* Δ*hpt* (Mm900) (Moore and Leigh, 2005), *Mc. maripaludis* Δ*hpt*Δ*earA* (Δ*earA*, Ding et al., 2016b) and mutant strains derived from them were routinely cultured in 120 mL sealed serum bottles containing 10 mL Balch medium III under a headspace of H_2_:CO_2_ (80:20) with shaking at 35°C (Balch et al., 1979). *Escherichia coli* TOP10 cells were cultured in Luria-Bertani (LB) broth or LB agar in the presence of 100 μg/mL ampicillin for plasmid selection at 37°C. Strains used in this study are listed in **Table 1**.

**Table 1:** Strains and plasmids used in this study.

Strains or plasmids	Description	Reference
***Methanococcus maripaludis* strains**		
Mm900	*Mc. maripaludis* S2 Δ*hpt*, wild-type strain in this study	Moore and Leigh, 2005
Δ*earA*	Mm900 Δ*earA_Mma_*	Ding et al., 2016b
Δ*earA-sp*	A spontaneous mutant derived from Δ*earA* in which the transcription of *flaB2* was restored	This study
Δ*3A*	A mutant created from Δ*earA* in which three adenines were deleted from the BRE region of the *fla* promoter	This study
***Escherichia coli* strains**		
TOP10	F^-^ *mcrA Δ*(*mrr-hsdRMS-mcrBC*) *φ80lacZΔM15 ΔlacX74 nupG recA1 araD139 Δ*(*ara-leu*)*7697 galE15 galK16 rpsL*(Str^R^) *endA1* aaa^-^	Invitrogen
**Plasmids**		
pCRPrtNeo	*hmv* promoter-*hpt* fusion plus Neo^r^ cassette in pCR2.1Topo; Amp^r^	Moore and Leigh, 2005
pKJ1273	pCRPrtNeo containing ∼2 kb region from *fla* promoter in which three adenines in the BRE region were deleted	This study

### Identification of a Spontaneous Mutant Strain (Δ*earA-sp*) Derived from Δ*earA* in Which the Expression of FlaB2 Was Restored

Immediately after its generation, the Δ*earA* mutant was streaked three times for purity, and one colony was grown overnight and frozen as the stock culture at -80°C. Western blot analysis confirmed the cessation of FlaB2 expression in the Δ*earA* strain at this stage (Ding et al., 2016a). The Δ*earA* strain was also maintained in the lab via weekly subculture in Balch medium III statically at 37°C. After 6 months of sub-culturing, western blotting experiments revealed that the expression of FlaB2 was restored. PCR experiments determined that this strain still had the deletion of *earA,* so the restoration of FlaB2 expression was not a result of strain contamination. The newly isolated strain was named as Δ*earA-sp* (*sp* for spontaneous).

### Sequence Analysis of the *fla* Promoter Region in the Δ*earA-sp* Strain

The *fla* promoter region spanning from -348 bp upstream of the TSS of the *fla* promoter to 162 bp downstream of the TSS from the Δ*earA-sp* strain was PCR amplified using primer pair P1-For/P1-Rev (**Table 2**) and washed Δ*earA-sp* cells as template (Ding et al., 2016b). The sequence of the PCR products was aligned with the corresponding region of the *Mc. maripaludis* S2 genome (NCBI version CAF31274.1) using Clustal Omega to detect the presence of any mutation (Goujon et al., 2010; Sievers et al., 2011).

**Table 2:** Primers used in this study.

Primers	Sequence	Restriction site incorporated (underlined)
**Promoter-substitution primers**	
P-fus-F	AGTCGGATCCATACATCAGTTTGACAGGAC	BamHI
P-fus-R	GACTGGATCCCAGCAAATGATGCATTAACG	BamHI
**Sequencing primers for promoter-substitution mutant screening**	
P1-For	TTTATAGATTCTGGATGTTCAAATGC	
P1-Rev	ATCAAGGTACCAATTCCAGAAGC	
earA-seq-F	TGGATACGGTAAGTTCCATCG	
earA-seq-R	CAACTTCGAGAATAGTGTCTCC	
**qRT-PCR primers**		
B2-qRT-PCR-For	GCTGCAATAGACATGAATCAGG	
B2-qRT-PCR-Rev	GACCAGTTTACAGTTGTAGTGTTG	
slp-qRT-PCR-For	GGTACTGAAGCATACGAAGGAG	
slp-qRT-PCR-Rev	GCTACAACTTTACCGTCTTTTAAGAG	

### Construction of Plasmids Used for the Δ*3A* Mutant Strain Generation

A mutant strain harboring the same three adenine deletion in the *fla* promoter BRE region as found in the Δ*earA-sp* strain was generated in the Δ*earA* mutant that showed no production of FlaB2 by western blotting. Briefly, an ∼2 kb DNA fragment containing the *fla* promoter region missing the three adenines in the BRE was PCR amplified with primers P-fus-F and P-fus-R (**Table 2**) and washed Δ*earA-sp* strain cells as template. The PCR product was digested with BamHI and cloned into BamHI digested pCRPrtNeo (Moore and Leigh, 2005) to create plasmid pKJ1273. Sequencing of the insert in pKJ1273 confirmed the three adenine deletion in BRE and no other changes. To generate the Δ*3A* mutant strain, pKJ1273 was transformed into Δ*earA* using a PEG-based method (Tumbula et al., 1994). The transformation mixture was cultured overnight without selection and then sub-cultured in McCas medium containing 1 mg/ml of neomycin for selection of cells in which pKJ1273 was integrated into the genome. After two passages in medium with neomycin selection, cells were cultured in McCas medium without neomycin to allow a second recombination event that would excise the pCRPrtNeo vector backbone, and this culture was plated onto McCas agar with 250 μg/mL 8-azahypoxanthine to kill any cells in which the vector backbone had remained integrated. Single colonies were picked and cultured in Balch medium III for western blot analysis of FlaB2 expression. For colonies in which the FlaB2 expression was restored, PCR was conducted to amplify both the *earA* gene region and the *fla* promoter region using primers listed in **Table 2** and washed cells as template. The size of the PCR amplicons of the *earA* gene region was analyzed by electrophoresis through 0.8% agarose gels to confirm the deletion in *earA*. The PCR products of the *fla* promoter region from seven colonies that produced FlaB2 and four colonies that did not produce FlaB2 were sequenced. One of the colonies that produced FlaB2 and contained the deletion of the targeted three adenines in the BRE region was restreaked for purity and designated as Δ*3A*.

### Western Blot Analysis of FlaB2 Expression in *Mc. maripaludis* Strains

The presence of the archaellin FlaB2 in the wild-type and various mutant strains of *Mc. maripaludis* was analyzed by western blot with an anti-FlaB2 antibody as previously described (Chaban et al., 2007).

### Quantitative RT-PCR (qRT-PCR) Analysis of the *flaB2* Transcription Level in *Mc. maripaludis* Strains

Total RNA from an *Mc. maripaludis* overnight cell culture was extracted using a High Pure RNA Isolation Kit (Roche Life Science) following a modified Gram negative bacteria RNA extraction protocol with an additional DNase treatment using a TURBO DNA-free Kit (Ambion) at 37°C for 30 min. Ten nanograms of total RNA from each extraction was converted into cDNA using an iScript^TM^ cDNA Synthesis Kit (Bio-Rad) with random hexamer primers. To detect the transcript level of *flaB2*, gene specific primers were constructed to amplify *flaB2* and the *slp* gene that encodes the S-layer protein (the latter was used as the reference) [**Table 2**, (Ding et al., 2016a)]. qRT-PCR experiments were performed as previously described (Ding et al., 2016a). Triplicates were included in each experiment, and three biological repeats were conducted.

### Swarming Motility Analysis of *Mc. maripaludis* Strains on Semi-Solid Agar

Five microliters of overnight cell cultures of each *Mc. maripaludis* strain (OD_600_ normalized to 1.0) grown in Balch Medium III were stabbed into Balch Medium III plates containing 0.25% agar (w/v) (Ding et al., 2015). Plates were incubated anaerobically in a canister under an atmosphere of H_2_:CO_2_ (80:20) at 37°C for 4 days.

### Electron Microscopy Analysis of *Mc. maripaludis* Strains

Cells grown overnight in Balch medium III were centrifuged and the pellets washed briefly with 2% NaCl (w/v), and resuspended in 2% NaCl. Cell resuspensions were loaded on 200-mesh carbon-coated copper grids. After adhesion to the grid for 1 min, cells were washed with 2% NaCl and then stained with 2% (w/v) phosphotungstic acid, pH 7.0. Samples were examined with a Philips CM-10 transmission electron microscope at 80 kV and images were taken with a SIS/Olympus Morada 11-megapixel charge-coupled device camera under standard operating conditions.

## Results

### Isolation and Identification of a Spontaneous Mutant of the Δ*earA* Strain in Which FlaB2 Expression Was Restored

**FIGURE 1:**
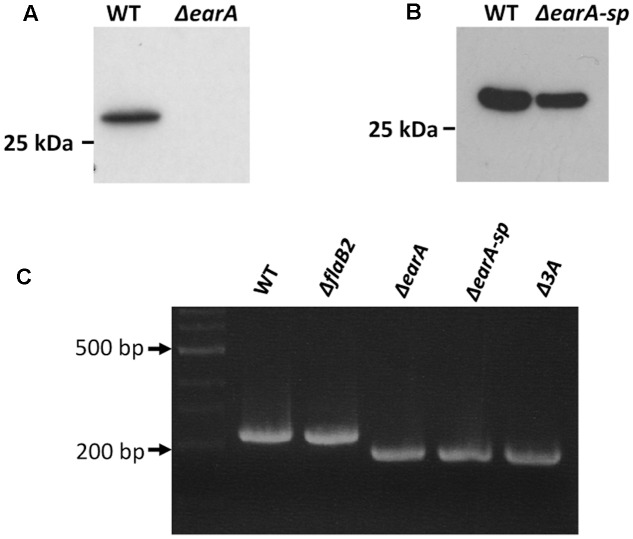
Western blot analysis of wild-type cells, the Δ*earA* strain and a spontaneous mutant of the Δ*earA* strain*, ΔearA-sp,* in which the expression of FlaB2 was restored. **(A)** Wild-type cells (Mm900) produce FlaB2 readily detected in western blots using FlaB2-specific antibodies. In the Δ*earA* strain, where the gene encoding the transcriptional activator EarA required for the transcription of the *fla* operon has been deleted, no FlaB2 was detected. **(B)** In the spontaneous mutant Δ*earA-sp*, the expression of FlaB2 was restored. **(C)** Confirmation of the deletion of *earA* in Δ*earA-sp* and Δ*3A* mutants. PCR products obtained using Δ*earA*, Δ*earA-sp* and Δ*3A* mutant cells as templates with primers amplifying the flanking area of the *earA* gene were smaller than those obtained using wild-type and Δ*flaB2* cells as template with the same primer pair, confirming that *earA* was deleted in the Δ*earA-sp* and Δ*3A* mutants.

In *Mc. maripaludis*, the transcription of the *fla* operon is dependent on the transcription activator EarA (Ding et al., 2016b). In the absence of EarA, as in the Δ*earA* strain, the archaellin FlaB2 (encoded by the second gene in the *fla* operon) is not detected in western blots (**Figure 1A**) and cells are non-archaellated. However, continuous weekly transfer of the Δ*earA* strain for about 6 months resulted in the isolation of a mutant form of the Δ*earA* strain in which FlaB2 synthesis was restored (**Figure 1B**). This spontaneous mutant was designated as Δ*earA-sp*. The deletion of the *earA* gene in Δ*earA-sp* was still present, as confirmed by PCR analysis of this strain compared to the original Δ*earA* strain and Mm900 cells. As shown in **Figure 1C**, both Δ*earA* and the Δ*earA*-*sp* cells had the expected smaller amplicon size obtained in PCR using primers flanking the deletion area of *earA* compared with amplicons obtained using Mm900 or Δ*flaB2* cells as template, ruling out the possibility that the restoration of FlaB2 in Δ*earA*-*sp* was due to contamination with the wild-type Mm900 strain or any other *Mc. maripaludis* strain with an intact *earA*.

**FIGURE 2:**
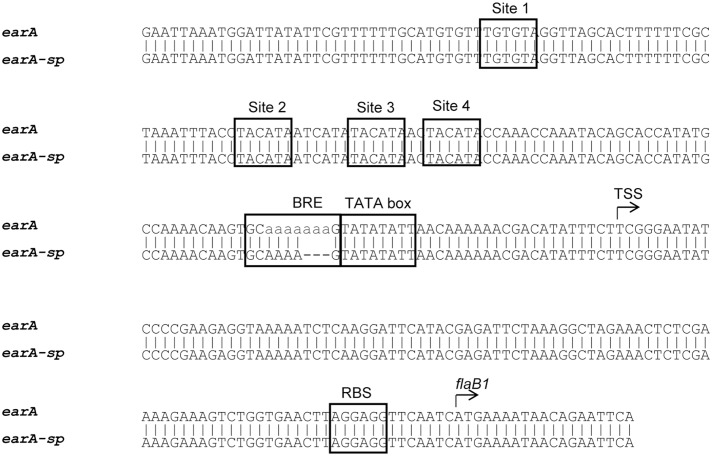
DNA sequence alignment of the *fla* promoter region from the Δ*earA* and Δ*earA-sp* strains showed a three adenine deletion in the BRE region. The four EarA binding sites (sites 1–4), BRE, TATA box, and ribosome binding site (RBS) are boxed in the figure. The transcription start site (TSS) and the start codon of the first gene in the *fla* operon, *flaB1*, are also indicated.

As an initial step in an effort to determine how these cells had regained the ability to transcribe the *fla* operon genes without EarA, we amplified and sequenced a ∼500 bp region encompassing the *fla* promoter from Δ*earA-sp* [from -348 nt to +162 nt with respect to the TSS; (Ding et al., 2016b)]. Analysis of the sequencing data showed that the four EarA binding sites (Ding et al., 2016b) upstream of the *fla* promoter remained intact, as did the TATA box, but in a stretch of seven adenines in the BRE found immediately upstream of the TATA box, three out of the seven adenines were missing in the Δ*earA-sp* strain (**Figure 2**). No other changes were found in the sequence of the PCR product amplified from the *fla* promoter region in the Δ*earA-sp* strain.

### Construction of a Δ*3A* Mutant in Which the Three Adenine Deletion in the BRE Was Recreated

**FIGURE 3:**
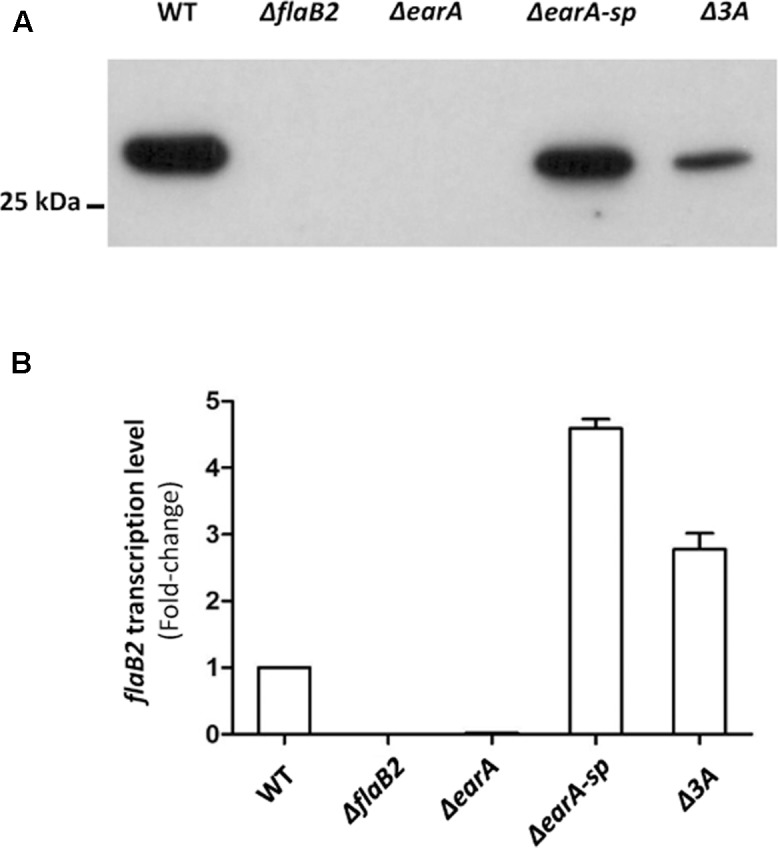
The translation and transcription of *flaB2* was restored in the Δ*earA-sp* and Δ*3A* mutants. **(A)** Western blot analysis showed that FlaB2 was expressed in both Δ*earA-sp* and Δ*3A* mutants, as well as in the wild-type cells but not in the Δ*earA* mutant or in a mutant deleted for *flaB2*. **(B)** The transcription of *flaB2* was restored in Δ*earA-sp* and Δ*3A* strains, as detected by qRT-PCR experiments. While transcripts for *flaB2* were barely detectable in the Δ*earA* mutant, *flaB2* transcription in the Δ*earA-sp* and Δ*3A* strains exceeded that of wild-type cells. Error bar shows standard derivation from nine data sets from three biological repeats, each of which were performed with triplicates.

It is possible that mutations other than the three adenine deletion in the *fla* promoter region could have occurred elsewhere in the genome of Δ*earA-sp* that were solely, or partially, responsible for the restoration of FlaB2 production. To explore if the three adenine deletion detected in the *fla* promoter region in the Δ*earA-sp* strain alone would result in the restoration of expression of FlaB2 in the absence of EarA, a mutant which carried the same three adenine deletion mutation in the *fla* promoter region as that in Δ*earA*-*sp*, was generated from the original stock Δ*earA* strain that did not synthesize FlaB2. Since the size difference in the *fla* promoter region of Δ*earA* and the generated three adenine mutant would be only three nucleotides, we did not try to screen mutants by PCR analysis. Instead, we used western blotting to screen for FlaB2 production, since if the deletion of the three adenines was responsible for restoration of transcription of the *fla* operon, transformants bearing this deletion would be readily identified from transformants that had retained the wild-type seven adenine sequence in the BRE region. Western blotting of a random number of transformant colonies appearing on 8-azahypoxanthine plates identified both ones that did and did not synthesize detectable amounts of FlaB2. The sequence of the *fla* promoter of four colonies where FlaB2 production was detected and seven colonies in which FlaB2 production was not detected were determined. In each of the colonies in which no FlaB2 was detected by western blotting, a wild-type *fla* promoter sequence, i.e., with seven consecutive adenines in the BRE, was found. In each of the four colonies that were found to produce FlaB2, the *fla* promoter was identical to the wild-type sequence except for the three adenine deletion in the BRE (data not shown). One of the transformant colonies that produced FlaB2 and had the three adenine deletion in the BRE was designated Δ*3A* and studied further. As shown in **Figure 3A**, FlaB2 production in the Δ*earA*-*sp* strain was near wild-type levels. In contrast, in the Δ*3A* cells, the expression level of FlaB2 was lower than that from the Δ*earA-sp* strain. PCR analysis of the Δ*3A* cells confirmed that these cells still possessed the deletion in *earA* (**Figure 1C**).

### Transcription of *flaB2* in the Δ*earA-sp* and Δ*3A* Strains Was Restored

Restoration of FlaB2 synthesis in the Δ*earA-sp* and Δ*3A* strains as demonstrated by the western blot results indicated that transcription of *flaB2* was occurring in both mutant strains. A direct measure of the transcript level of *flaB2* in these two mutants as well as control strains was obtained in qRT-PCR experiments (**Figure 3B**). As expected, *flaB2* transcripts were not detected in the Δ*flaB2* strain and were barely detectable in the Δ*earA* strain. In contrast, the transcription level of *flaB2* was increased over 4-fold and 2.5-fold in the Δ*earA-sp* and the Δ*3A* strains, respectively, compared to that detected in wild-type cells. The relatively higher transcription level of *flaB2* in the Δ*earA*-*sp* cells compared to the Δ*3A* cells was consistent with production of FlaB2 in the two strains detected in the western blot. However, the production of FlaB2 in the Δ*3A* cells was lower than in wild-type cells even though *flaB2* transcription was higher.

### Δ*earA-sp* and Δ*3A* Strains Were Archaellated

**FIGURE 4:**
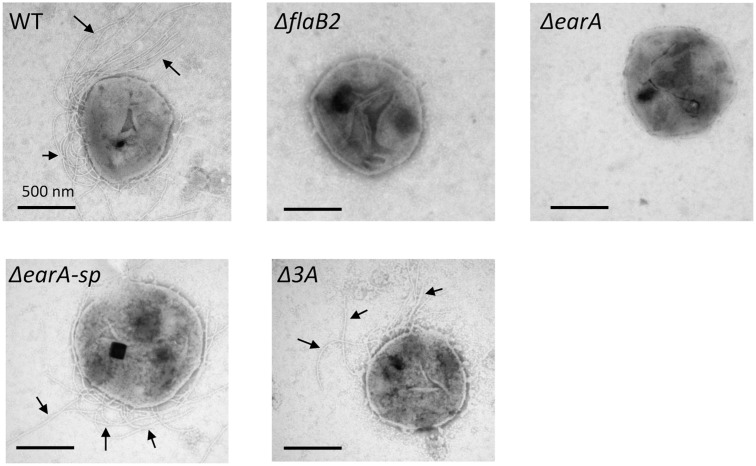
Electron micrographs illustrating archaella on the surface of Δ*earA-sp* and Δ*3A* mutants, as well as wild-type cells. As expected, the Δ*flaB2* and Δ*earA* mutants were non-archaellated. Bars equal 500 nm.

qRT-PCR and western blot analyses demonstrated that transcription and translation of *flaB2* had been restored in the Δ*earA*-*sp* and Δ*3A* strains. To determine if the transcription and translation of the entire *fla* operon was restored in the two mutant stains resulting in assembly of archaella, cells were examined by electron microscopy. As shown in **Figure 4**, archaella were observed on the cell surface of both Δ*earA-sp* and Δ*3A* cells, as well as the wild-type cells, but not on Δ*flaB2* or Δ*earA* cells.

### Δ*earA-sp* and Δ*3A* Strains Had Swarming Motility

**FIGURE 5:**
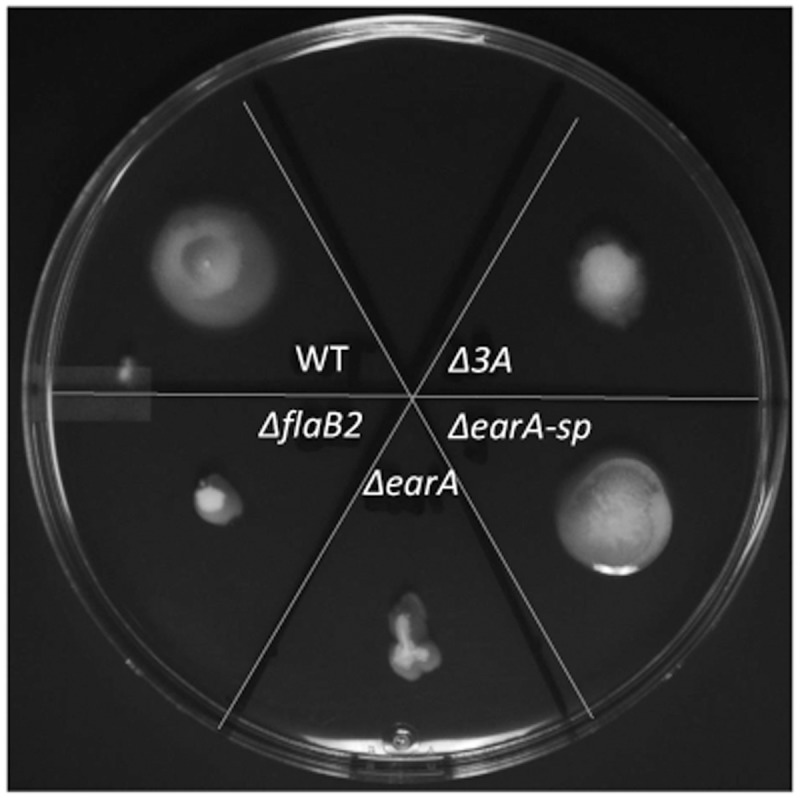
Swarming assay demonstrating the motility of the Δ*earA-sp* and Δ*3A* mutants. Overnight cell cultures were normalized with respect to their OD_600_ and the same amount of cells were inoculated onto Balch medium III plates containing 0.25% agar and incubated for 4 days at 37°C.

To further determine if the archaella observed on Δ*earA-sp* and Δ*3A* cells were functional, swarming motility assays were performed. Overnight cultures of Δ*earA-sp,* Δ*3A*, as well as Mm900, Δ*flaB2*, and Δ*earA* strains were inoculated onto semi-solid Balch medium III agar. After incubation at 37°C for 4 days Mm900, Δ*earA-sp* and Δ*3A* cells were clearly motile although the motility of the Δ*3A* cells was less than the other two strains (**Figure 5**). The non-archaellated strains, Δ*flaB2* and Δ*earA*, remained at the inoculation spot, as expected. The swarming data are consistent with data from western blot, qRT-PCR, and EM analyses.

## Discussion

Previous studies have shown that the euryarchaeal archaellum regulator EarA was critical for transcription of the *fla* operon in *Mc. maripaludis* via its binding to at least one of four consensus sequences located immediately upstream of the BRE and TATA box of the *fla* promoter. In a Δ*earA* mutant, transcription of the *fla* operon is barely detectable and cells are non-archaellated (Ding et al., 2016b). In this study, we have isolated a spontaneous mutant of a Δ*earA* strain in which the transcription of the *fla* operon and archaellation were restored. Analysis of the DNA sequence of the *fla* promoter region in this mutant, designated Δ*earA-sp,* revealed a deletion of three adenines in the BRE region. Recreation of the three adenine deletion in the stock strain of the Δ*earA* mutant also led to restoration of *fla* operon transcription and archaellation, indicating that this small deletion in the BRE overcame the requirement for EarA for activation of transcription of the *fla* operon. However, the expression of FlaB2 detected by western blotting was lower in the recreated strain than in the spontaneous mutant Δ*earA-sp,* suggesting that the three adenine deletion may not be the sole change in the Δ*earA-sp* strain affecting transcription of the *fla* operon. However, it seems clear from our studies on the directed mutant Δ*3A* strain, that the deletion of three adenines in the BRE of the *fla* operon promoter is sufficient on its own to result in all the phenotypes related to archaellation observed in the spontaneous mutant.

Since there is virtually no transcription detected from the native *fla* promoter if *earA* is deleted, it suggests that the *fla* promoter is intrinsically very weak or inactive. Two key elements that determine promoter strength in Archaea are the sequences of the TATA box and BRE (Bartlett, 2005). The TATA box is the site of binding of the TATA-binding protein TBP while the BRE sequence is the site of binding for TFB (Peeters et al., 2013). While relatively few transcriptional activators have been studied in Archaea, the mechanism of activation in these limited studies has been shown to involve recruitment of TBP or TFB to the TATA box or BRE (Karr, 2014). Consensus TATA box sequences vary for different subgroups of Archaea and mutations in the TATA box can reduce transcription efficiency (Soppa, 1999; Bartlett, 2005; van de Werken et al., 2006). For protein promoters in *Mcc. jannaschii*, the TATA box was determined to be TWTATATA (where W = A or T) (Zhang et al., 2009), very similar to the TTTATATA proposed previously for the promoters of stable RNA genes in *Methanococcus vannielii* (Thomm and Wich, 1988) and featuring the methanogen characteristic of strict alterations of T and A in contrast to TATA boxes in other major archaeal groups (Soppa, 1999). One of the best-studied archaeal transcriptional activators, Ptr2 of *Mcc. jannaschii*, binds to multiple sequences upstream of BRE in the rubredoxin 2 gene and has its stimulatory effect due to direct recruitment of TBP (Ouhammouch et al., 2003, 2005). Adding binding sites for Ptr2 upstream of heterologous promoters with sub-optimal TATA box sequences resulted in significant transcriptional activation (Ouhammouch et al., 2005). Analysis of the TATA box of the *fla* operon in *Mc. maripaludis* revealed a strong identity to the consensus sequence, including the alternating T and A stretch TATATAT, suggesting binding of TBP should not be impaired.

**FIGURE 6:**
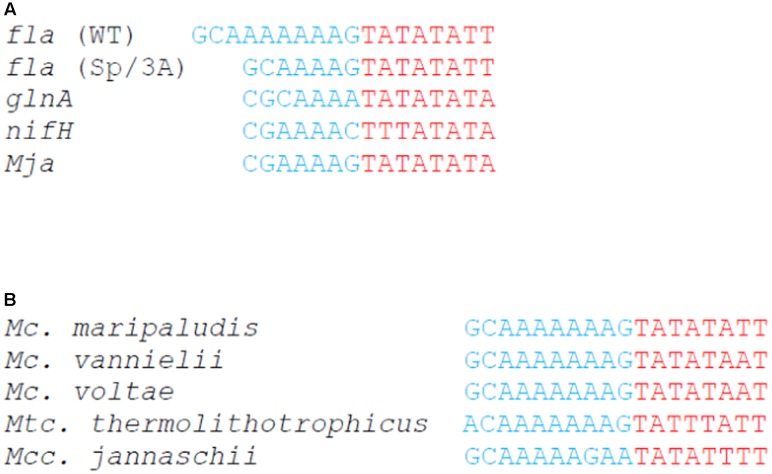
Promoter sequence analysis of the wild-type and mutated *fla* promoters and other archaeal promoters. **(A)** Promoter sequences of *fla* promoter (*fla*), mutated *fla* promoter with the three adenine deletion in the BRE (Sp/3A), *glnA* promoter (glnA), and *nifH* promoter (nifH) from *Methanococcus maripaludis*, as well as the conserved promoter sequence from the tRNA^lys^ gene of *Methanocaldococcus* *jannaschii* (Mja). **(B)** BRE/TATA box sequences of the *fla* operon promoters of selected Methanococcales.

The 6–7 nucleotide long BRE sequences are the major site of binding for TFB, with positions -3 and -6 of BRE (relative to the TATA box) showing the strongest specificity determinants (Qureshi and Jackson, 1998; Littlefield et al., 1999). There are no BRE consensus sequences reported for halophiles and methanogens (van de Werken et al., 2006). However, in *Mcc. jannaschii*, a hyperthermophilic relative of *Mc. maripaludis*, two studies have identified promoter sequences on a whole genome basis (Li et al., 2008; Zhang et al., 2009). The first study used the binding of TBP and TFB in EMSA studies to identify promoters (Li et al., 2008). These studies had a strong bias for strong promoters, especially for promoters of tRNA genes with only small percentage of promoters for protein genes being retrieved. These studies led to the identification of an extended BRE element sequence of 9–10 nucleotides (MRCCGAAAAG where M = A, C and R = A, G). The second study focused on identification of promoters for protein-encoding genes (Zhang et al., 2009). It was found for *Mcc. jannaschii* protein gene promoters that there was a greater variability in the BRE than in the TATA box (Zhang et al., 2009). The identified promoters for protein-encoding genes were shown to bind the general transcription factors less tightly than tRNA gene promoters. Notably, base frequencies at several BRE positions considered important for TFB binding were significantly different from the *in vitro* selected promoters (mostly for tRNA genes) in the earlier study. Examination of the BRE sequences in both protein-encoding genes and tRNA genes revealed that most had internal stretches of 3–5 adenines, far less than the seven adenines in the wild-type *fla* operon promoter. Interestingly, the *fla* operon BRE element has a G at position -1, the most commonly found base at that position in the strong tRNA gene BRE (**Figure 6A**), while in protein-encoding genes the most common base at -1 is C (Zhang et al., 2009).

The wild-type version of the BRE of the *fla* operon promoter, with its stretch of seven adenines, does not show strong sequence identity to what may be considered strong BRE sequences as reported for *Mcc. jannaschii*. On the other hand, it is apparent that the three adenine deletion version found in the Δ*earA-sp* mutant much more closely aligns with BRE sequences found in strong tRNA gene promoters of *Mcc. jannaschii* (**Figure 6A**). In addition, the mutated *fla* promoter in the Δ*earA-sp* strain shares high sequence identity with two studied promoter sequences in *Mc. maripaludis*, namely the nitrogen-regulated *glnA* and *nifH* promoters (**Figure 6A**) (Cohen-Kupiec et al., 1997, 1999). Both *glnA* and *nifH* promoters are regulated via the repressor NrpR, which binds to the *nif* operators located downstream of the TATA boxes just after the TSS in the two promoters leading to repression of transcription under ammonia growth conditions (Cohen-Kupiec et al., 1997, 1999; Lie et al., 2005). Both *nifH* and *glnA* expression is very low when cells are grown on ammonia and NrpR binds but high expression is observed under conditions of diazotrophic growth where NrpR does not bind or in a strain where *nrpR* has been deleted (Cohen-Kupiec et al., 1997, 1999; Lie and Leigh, 2003; Lie et al., 2005). This indicated that the basal transcription level of the two promoters was strong, suggesting that TFB and TBP in *Mc. maripaludis* could recognize BRE and the TATA box of these two promoters and initiate transcription (Cohen-Kupiec et al., 1997, 1999). The high sequence identity of the three adenine deletion BRE of the Δ*earA-sp* strain with that of the *glnA* and *nifH* promoters, as well as the BRE of the highly expressed tRNA genes of *Mcc. jannaschii* likely explains why the pre-initiation complex could be formed with the mutated *fla* promoter without the aid of EarA. The qRT-PCR results (**Figure 3B**) suggest that the wild-type *fla* operon promoter even with EarA is not as strong as the three adenine deletion version in the absence of EarA.

Studies in several archaea have indicated that promoters containing non-conserved BRE sequences can be weak or even inactive (Peng et al., 2009, 2011; Marschaus and Pfeifer, 2012; Ochs et al., 2012). Replacement of the BRE of inducible promoters with a BRE from strong promoters, for example, can greatly increase the transcription from the resulting chimeric promoter. In *Sulfolobus solfataricus,* transcription from the arabinose promoter is induced in the presence of arabinose, via an unidentified factor that binds to a consensus ara-box sequence located immediately upstream of the BRE and TATA box (Lubelska et al., 2006; Peng et al., 2011). When the BRE from the arabinose promoter was replaced with the strong BRE from the *Sulfolobus shibatae* viral (SSV) T6 promoter (Qureshi and Jackson, 1998), the resulting chimeric promoter was now constitutive and not regulated by the ara-box element (Peng et al., 2009). The apparent mechanism of transcription activation of the ara-box binding factor is thought to be by recruitment of TFB to a weak BRE (Peng et al., 2009, 2011). In *Pyrococcus furiosus*, transcription from the *pf1089* promoter is activated by PF1088 (Transcription factor B recruitment factor 1, TFB-RF1). This activation is dependent on the weak BRE of the *pf1089* promoter and is not observed if the *pf1089* promoter BRE is replaced with the BRE from the strong *gdh* promoter (Spitalny and Thomm, 2003; Ochs et al., 2012). Electrophoretic mobility shift assays further revealed that the transcription activation of the wild-type *pf1089* promoter was by the recruitment of TFB via TFB-RF1, thereby overcoming the weak BRE.

We have recently shown that EarA homologs are commonly found in the Euryarchaota and that EarA proteins from numerous methanogens can rescue the defects in archaellation in a *Mc. maripaludis ΔearA* strain (Ding et al., 2017). As shown in **Figure 6B**, examination of the *fla* promoter regions in selected archaellated Methanococcales containing an *earA* homolog revealed BRE sequences identical, or very similar, to that in *Mc. maripaludis*, i.e., with a string of seven adenines as in *Methanococcus voltae*, *Methanococcus vannielii*, and *Methanothermococcus thermolithotrophicus* or seven adenines in a stretch of eight nucleotides in the BRE of the *fla* promoter of *Mcc. jannaschii*. It would appear that in all these cases, the *fla* promoter requires the presence of EarA proteins to overcome weak BRE sequences, presumably to aid in the recruitment of TFB, as found for transcriptional activators TFB-RF1 and the ara-box binding factor.

The appearance of the Δ*earA-sp* mutant was surprising to us. The isolation of the original Δ*earA* mutant arose after it was discovered that, after repeated transfers in the lab, mutants carrying deletions of various *fla* or *agl* genes required for assembly of archaella stopped transcription of the *fla* operon and the *fla* operon reporter protein FlaB2 could not be detected in western blots (Vandyke et al., 2009; Ding et al., 2016b). It was determined that in at least some of these mutants, the reason for the cessation of *fla* operon transcription was a reading-frame shift mutation in *earA*. We reasoned that in these strains that carried mutations in *fla* or *agl* genes necessary for archaella assembly, it was an advantage to no longer synthesize several proteins, some of which, like archaellins, were required in large amounts when they could not be assembled in archaella. This led to a selective advantage for those cells that had stopped transcription of the *fla* operon, as in the *earA* mutants. Thus, it is not obvious to us why a mutation in the Δ*earA* that would restore transcription of the *fla* operon would arise and outgrow the original Δ*earA* mutant. The answer may lie in the presence of an additional mutation(s) in the Δ*earA-sp* strain that could be revealed by comparison of the complete sequence of the Δ*earA* and Δ*earA-sp* strains.

In this study, a spontaneous mutant with restored FlaB2 expression was isolated from a Δ*earA* mutant, indicating that in the spontaneous mutant the need for the transcriptional activator EarA for the transcription of the *fla* promoter was bypassed. Analysis of the DNA sequence in the *fla* promoter region from the spontaneous mutant revealed a three adenine deletion in the BRE region in the *fla* promoter. Sequence alignment showed that the mutated BRE in the *fla* promoter shares high similarity with BREs from strong promoters in methanogens, indicating that with this mutated BRE, the transcription initiation of the *fla* promoter could be conducted with components of the basal pre-initiation complex. We believe this is the first report of spontaneous mutation in the promoter region that overcomes the need for a transcriptional activator and it emphasizes the key role played by the BRE in promoter strength in Archaea.

## Author Contributions

Conceived and designed experiments: YD, AB, CK, and KJ. Performed the experiments: YD, AB, CK, and KJ. Analyzed the data: YD, AB, CK, and KJ. Wrote the paper: YD, AB, CK, and KJ.

## Conflict of Interest Statement

The authors declare that the research was conducted in the absence of any commercial or financial relationships that could be construed as a potential conflict of interest.
